# Harrison Narcotic Law*Read before North Texas Medical Association, December 13, 1916.

**Published:** 1917-02

**Authors:** W. E. Allen

**Affiliations:** Assistant United States Attorney


### TEXAS MEDICAL JOURNAL

DR. F. E. DANIEL, Founder.

MRS. F. E. DANIEL, Publisher and Managing Editor.

PUBLISHED MONTHLY. Subscription: $1.00 a Year.

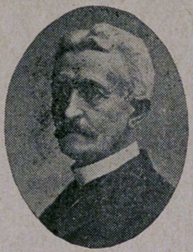

Established July, 1885.

VOL. XXXII No. 8
AUSTIN, FEBRUARY, 1917.

The publisher is not responsible for views of contributors.

*"All of good the past hath had,
Remains to make our own time glad."*—WHITTIER.

### Original Articles.

### Harrison Narcotic Law.*

BY W. E. ALLEN, ASSISTANT UNITED STATES ATTORNEY.

We are living under a dual form of government, each in a measure separate and distinct from the other. The States are possessed of the power to pass all laws not prohibited by the Constitution of the United States. The lawmaking body of the United States may pass only such laws as it is specifically authorized to pass by the Constitution. In other words, the Federal government is one of limited legislative power. The Congress can act in those matters only where it is authorized to act by the Constitution of the United States.

Among the powers thus delegated to Congress is the right to raise revenue for the maintenance of the government, and to pass suitable laws providing for the collection of such revenue and to prevent any fraud upon the revenue. It is this provision of the Constitution that gave Congress the power to pass the Act of December 17, 1914, commonly known as the Harrison Anti-Narcotic Law.

It is quite apparent that Congress had in mind more far-reaching consequences than the mere raising of revenue. Our national lawmakers, with the sad history of the most populous nation of the world before them, where the very vitality of that

*Read before North Texas Medical Association, December 13, 1916.

nation has been sapped by the use of opium, and with the knowledge that the drug is finding its way into this country to an alarming extent, sought to check the evil at the very outset. Thus, the most drastic law classed as a revenue measure that has ever found its place on the statute books of the United States.

By the first section of this law it is provided that every person who produces, imports, manufactures, compounds, deals in, dispenses, sells, distributes, or gives away opium or coca leaves or their derivatives, shall pay a tax and shall register with the collector of internal revenue. In subsequent sections of the act, it is required that persons growing, importing, or handling these drugs in any way, shall keep a record thereof. In this way it is quite evident that the government proposes to keep a check on all narcotic drugs produced in the United States or brought into the United States from any foreign country. Congress had a right to require such a record to protect the revenue, which quite satisfies the constitutional requirement and gives to the law its validity.

Section 2 of the act provides that whoever shall sell, barter, exchange, or give away any of the drugs specified in the act shall do so in pursuance of a written order, which order shall be in duplicate, the form of the order being prepared by the Revenue Department.

There are certain exceptions to this requirement, and it is with these exceptions you are particularly concerned, as they relate to the acts of a physician. The first exception exempts from the requirement the dispensing or distribution of any of the said drugs by a physician, dentist or veterinary surgeon to a patient in the course of the professional practice of such physician, dentist, or veterinary surgeon.

The inquiry then naturally arises, first, who is a patient? This matter was presented to the Hon. E. R. Meek, United States District Judge for the Northern District of Texas, at Dallas, in the trial of a case in January, who defined the word "patient" as follows: "'Patient,' as and wherever used in the charge of the court, gentlemen, means a person undergoing treatment for disease." The next inquiry is, What is the meaning of the term "professional practice only," or "legitimate practice of his profession," as used in the act? The court in the same case defined that term as follows: "'Legitimate practice of his profession,' as used herein, means regularly and properly undertaking by a physician, first, to judge the nature, character and symptoms of disease; second, to determine the proper remedy for the disease; and, third, to give, prescribe, or dispense the application of the remedy to the disease."

I know of no better or clearer definitions of these terms than those announced by the court. Therefore, a physician who is engaged in the legitimate practice of his profession may use narcotics as his judgment dictates in the treatment of any patient, and may do that without receiving from such patient a written order for the drug.

In this connection, however, it is required that the physician keep a record of all drugs dispensed or distributed by him, except such as may be dispensed or distributed to a patient upon whom such physician, dentist or veterinary surgeon shall personally attend. What does "personally attend" mean? The Honorable Commissioner of Internal Revenue, the head of the Revenue Department, has ruled that this section of the law requires a physician to keep a record of all drugs dispensed, used, or distributed by him, except such drugs as may be administered during a visit to a patient away from the office of the physician. This question, so far as I know, has never been determined by a court. At least, it has not been determined by the judge of this district, nor by any appellate court. That being the case, the ruling of the Honorable Commissioner of Internal Revenue should be given weight and should be accepted as a correct construction of the law. My advice to any physician is to keep a record of all drugs dispensed by him at his office.

Another question which has quite frequently arisen, and one that I have had propounded to me by a number of physicians, is as to the right of a physician to prescribe for an addict who is afflicted with an incurable disease. As I understand the law and the rulings of the Department, they are that a physician may prescribe such quantities of the drug to anyone suffering with disease as the good judgment of the physician deems necessary. In other words, if you have a patient suffering with cancer, tuberculosis, or some other incurable disease, and you think the use of narcotics is necessary in the treatment of the patient or the alleviation of pain, you are at liberty to prescribe for such patient the drug to be used in such quanties as the particular case may require. However, a prescription in that instance should show on its face the malady and also the dosage prescribed.

It is not intended by this law to hamper the legitimate practitioner of medicine in his work, nor is it intended to take from such physician the remedies which he considers necessary or proper in the practice of his profession. The law has placed upon the physician a great trust. He possesses special and superior knowledge of the subject, and, so long as he is acting in good faith, the law does not interfere. He must not abuse the trust thus confided in him, and the ethical practitioner will not abuse that trust. I know this from my experience of almost two years in dealing with this law and seeking to enforce it.

Just how far, if at all, a physician may go in prescribing for an addict not afflicted with any disease has not been determined. It seems quite clear, however, that the law does not contemplate the giving or prescribing of the drug to an addict simply to satisfy the cravings of such addict for the drug. In my judgment, such acts would fall within the condemnation of the law.

Whether or not the writing of prescriptions is dispensing the drug has not yet been definitely determined. One court has held that it is, but the Revenue Department has not ruled on that point, and for that reason has not seen fit to require that prescriptions shall be written in duplicate and a copy kept by the physician. However, a great many physicians do this. I have seen a number of forged prescriptions, and also prescriptions where the quantity of the drug prescribed had been materially increased by the person for whom prescribed. A copy of each prescription would be of aid to the government in detecting frauds perpetrated by those who receive prescriptions, but I do not understand that the Department has seen fit to require that a copy of each prescription be kept.

You are familiar with the form of prescription to be used when narcotics are prescribed. This form should be used in every instance where any quantity of a narcotic drug is mentioned in the prescription.

Section 6 of the law exempts from its operation certain proprietary remedies and preparations, and I regret to say that this section has been abused by a few druggists. It is made very clear by the terms of Section 6 that it shall not apply where the purpose is to evade the intentions and provisions of the act. In other words, druggists cannot supply addicts with quantities of colic cures and other remedies containing narcotics where it is apparent that the medicine is being used simply to satisfy the cravings of the purchaser. Druggists who sell excessive quantities of any preparation containing morphine or its derivatives are on dangerous ground, and while I am specifying drugs I should include here paregoric. Paregoric may be sold under Section 6 when sold as a medicine and in such quantities as the druggist may reasonably believe are being purchased as medicine, but when sold in excessive quantities the law is clearly violated. You perhaps have had trouble with some of your patients on account of the exceptions contained in Section 6. The Department would appreciate information at any time of any violation of this section. In other words, if you have a patient who is supplying himself with narcotics by purchasing excessive quantities of remedies containing narcotics, the Federal authorities would appreciate your advising them of that fact.

A druggist may dispense or distribute narcotic drugs on a bona fide prescription of a physician prepared in conformity with the law. One of the requirements is that the prescription shall be written and signed by the physician. This would seem to exclude the idea that a prescription given over the telephone would be sufficient. I know that it is the conception of the Revenue Department that such prescriptions should not be filled. We can realize that a situation could arise where such prescription could not be given and the drug procured thereon in time to answer the purpose. These occasions, however, are rare. Wherever it is possible for the drug to be procured in the regular way, the Department insists upon a compliance with the letter of the law. However, I do not say that in every instance where a druggist fills a prescription in an emergency case given to him by a physician whose voice he knows and where the physician prepares a prescription and delivers it to the druggist's messenger that the druggist would be prosecuted. If the practice of telephoning prescriptions should prevail generally, and such prescriptions be substituted for written prescriptions, there would be a field opened up to the unscrupulous. The law is intended to be construed with reason, and, as I have said, much depends upon the good faith of those prescribing or dispensing the drug.

This law is not perfect. It has its defects.

The first automobile invented was not perfect, but it formed the basis for a machine which is now well-nigh perfect. So it is with the Harrison Narcotic Law: it is the basis of other laws. I believe that physicians should study the law and should offer all suggestions possible looking to the remedying of all defects; and I am pleased to say that the physicians with whom I have come in contact, almost without exception, have been the best friends that the Harrison Narcotic Law has had.

